# Superficial white matter imaging: Contrast mechanisms and whole-brain in vivo mapping

**DOI:** 10.1126/sciadv.aaz9281

**Published:** 2020-10-07

**Authors:** Evgeniya Kirilina, Saskia Helbling, Markus Morawski, Kerrin Pine, Katja Reimann, Steffen Jankuhn, Juliane Dinse, Andreas Deistung, Jürgen R. Reichenbach, Robert Trampel, Stefan Geyer, Larissa Müller, Norbert Jakubowski, Thomas Arendt, Pierre-Louis Bazin, Nikolaus Weiskopf

**Affiliations:** 1 Department of Neurophysics, Max Planck Institute for Human Cognitive and Brain Sciences, Stephanstraße 1a, 04103 Leipzig, Germany.; 2 Center for Cognitive Neuroscience Berlin, Free University Berlin, Habelschwerdter Allee 45, 14195 Berlin, Germany.; 3 Paul Flechsig Institute of Brain Research, Leipzig University, Liebigstr. 19, 04103 Leipzig, Germany.; 4 Felix Bloch Institute for Solid State Physics, Faculty of Physics and Earth Sciences, Leipzig University, Linnéstraße 5, 04103 Leipzig, Germany.; 5 Medical Physics Group, Institute of Diagnostic and Interventional Radiology, Jena University Hospital–Friedrich Schiller University Jena, Philosophenweg 3, 07743 Jena, Germany.; 6 Department of Radiology University Hospital Halle (Saale), Ernst-Grube-Str. 40, 06120 Halle, Germany.; 7 Federal Institute for Materials Research and Testing, Richard-Willstätter-Straße 11, 12489 Berlin, Germany.; 8 Spetec GmbH, Berghamer Str. 2, 85435 Erding, Germany.; 9 Integrative Model-Based Cognitive Neuroscience Research Unit, University of Amsterdam, 1001 NK Amsterdam, The Netherlands.; 10 Wellcome Centre for Human Neuroimaging, Wellcome Centre for Human Neuroimaging, Institute of Neurology, University College London, 12 Queen Square, London WC1N 3AR, UK.

## Abstract

Superficial white matter (SWM) contains the most cortico-cortical white matter connections in the human brain encompassing the short U-shaped association fibers. Despite its importance for brain connectivity, very little is known about SWM in humans, mainly due to the lack of noninvasive imaging methods. Here, we lay the groundwork for systematic in vivo SWM mapping using ultrahigh resolution 7 T magnetic resonance imaging. Using biophysical modeling informed by quantitative ion beam microscopy on postmortem brain tissue, we demonstrate that MR contrast in SWM is driven by iron and can be linked to the microscopic iron distribution. Higher SWM iron concentrations were observed in U-fiber–rich frontal, temporal, and parietal areas, potentially reflecting high fiber density or late myelination in these areas. Our SWM mapping approach provides the foundation for systematic studies of interindividual differences, plasticity, and pathologies of this crucial structure for cortico-cortical connectivity in humans.

## Teaser

Superficial white matter mapping provides the foundation for systematic studies of this crucial structure.

## INTRODUCTION

Superficial white matter (SWM) is the thin layer of WM just underneath the cortical sheet. Its structure and function are substantially different from deep WM (DWM) and are strongly influenced by the proximity of the cortical gray matter (GM). SWM contains short association U-fibers that primarily connect adjacent gyri. These subcortical U-fibers represent most of the WM connections in the human brain (*1*) and are the last structures to be myelinated, maturing as late as the fourth or fifth decade of life. The important role of U-fibers in brain maturation, plasticity, and aging is reflected by the fact that reduced U-fiber density is observed in disorders such as autism (*2*), epilepsy (*3*), and Alzheimer’s disease (AD) (*4*).

The SWM also has a high density of interstitial WM neurons (*5*). With distinct inter-regional differences in density, the presence of these cells has challenged traditional views of SWM as a structure for passive information transfer. Neuronal circuits in SWM may modulate cortico-cortical connectivity by regulating the timing and signal transfer efficiency at the axonal connections (*6*).

Despite these proposals, surprisingly little is known about the structure, function, and metabolism of the SWM and the variation of U-fiber and interstitial neuron density across the human brain (*7*, *8*). One major reason for that is the lack of reliable SWM and U-fiber imaging methods. Gold-standard molecular tracer studies are not feasible in humans due to their invasiveness, while noninvasive fiber tractography, based on diffusion-weighted magnetic resonance imaging (MRI) (DWI), does not provide satisfactory results in SWM. Low spatial resolution of current DWI approaches can neither resolve the thin SWM layer nor disentangle the multitude of crossing fibers within it (*9*).

One promising method for in vivo SWM mapping is ultrahigh-resolution structural MRI. Recent advances in ultrahigh field MRI, combined with biophysical modeling of MR contrast, have enabled the imaging and mapping of specific aspects of brain microstructure (*10*). Important progress has been made in mapping the laminar structure and myelination patterns of the cortex (*11*, *12*) and fiber orientation-dependent WM contrast in DWM (*13*–*16*). However, no study has systematically applied microstructural imaging to SWM mapping yet.

One substantial obstacle is that the neurophysiological and biophysical mechanisms underlying MRI contrast in SWM are not well understood. It is now known that MR contrast in SWM (*17*) differs from that in DWM. In DWM, highly aligned myelinated axons, and their orientation, dominate MR contrast (*13*, *18*). Drayer *et al*. (*17*) have demonstrated enhanced transverse relaxation rates (*R2*) in SWM compared to DWM, in patients across different ages. By a visual comparison with postmortem Perls staining, the authors qualitatively linked the observed contrast to increased iron concentration in subcortical U-fibers. This observation was supported by Bagnato *et al*. (*19*) at 7 T who demonstrated hyperintense SWM on effective transverse relaxation rate (R2*) and phase maps in postmortem brain samples of patients with multiple sclerosis and controls. Other studies have also observed elevated iron levels in the SWM (*20*–*22*).

Although the contribution of iron to the susceptibility, *R2*, and R2* MRI contrasts in SWM has been established, it remains unclear which morphological microscopic structures influence the contrast, such as cell bodies or myelinated fibers. It is also not clear what the relative contributions of other tissue components are, such as myelin, to MRI contrast. Moreover, systematic whole-brain SWM mapping would require an understanding of the orientation dependence of MRI parameters within this structure with its complex geometry. A mechanistic understanding of the contrast mechanisms is therefore crucial for the interpretation of MRI parameters in SWM and the development of SWM mapping methods.

In this study, in order to lay the foundation for systematic SWM mapping, we aimed to establish a quantitative relationship between microstructure and MRI parameters. We studied several MR contrasts in SWM using high spatial resolution MRI in vivo and in human postmortem brain samples at 7 T. We show that *R2*, R2*, and quantitative susceptibility maps (QSMs) exhibit a strong contrast between SWM, cortical GM, and DWM. By comparing in vivo and postmortem MRI with postmortem quantitative iron maps, we identified iron as the dominant source of contrast in SWM. The microscopic iron maps were obtained by proton-induced x-ray emission (PIXE) and laser ablation inductively coupled plasma mass spectroscopic imaging (LA-ICP-MSI) at different spatial resolutions ranging from 1 to 100 μm. Furthermore, we developed a novel biophysical model that quantitatively links the iron-induced MR contrast to the microscopic iron distribution at the cellular level. Iron inside iron-rich oligodendrocyte bodies and other cellular tissue components was identified as the main source of susceptibility-related MR contrasts. In vivo imaging data show that the iron-induced contrast in SWM varies between different cortical brain areas. Increased iron deposits were observed in U-fiber–rich frontal, temporal, and parietal association areas, potentially reflecting higher fiber density or late myelination in these areas. This variability suggests a functional specificity of SWM and further supports the validity of susceptibility- and R2*-based markers.

The noninvasive mapping of *R2*, R2*, and quantitative susceptibility thus opens the door for systematic studies of the SWM in humans. Interregional variation, interindividual differences, and developmental trajectories of this important brain structure can now be investigated in terms of health and disease.

## RESULTS

The experiments described below followed a three-step approach. In a first step, we have empirically demonstrated that iron is the main contributor to MRI contrast in SWM. We have characterized the microscopic distribution of iron by combining in vivo and postmortem MRI with histological analyses and iron quantification methods. In a second step, we used the information about the meso- and microscopic iron distribution to develop models of MRI contrast in SWM. Two models were developed: an empirical linear model and a generative model. Both models combined iron and myelin as relaxation drivers, but the latter accounted for cellular iron distribution and orientation dependence of R2*. In a third step, we applied the generative model to map the iron distribution in SWM throughout the entire human brain in vivo.

### SWM shows increased values of magnetic susceptibility, *R2*, and R2* in line with elevated iron levels

High-resolution whole-brain (400-μm isotropic resolution) quantitative multi-parameter maps, including the longitudinal relaxation rate (*R1*), effective transverse relaxation rate (R2*), proton density map (PD), and magnetic susceptibility (χ), were obtained in vivo in four healthy human volunteers (*11*). Combined *R2* and R2* measurements (500-μm in-plane resolution) were performed in one slice on a fifth volunteer.

**Fig. 1:**
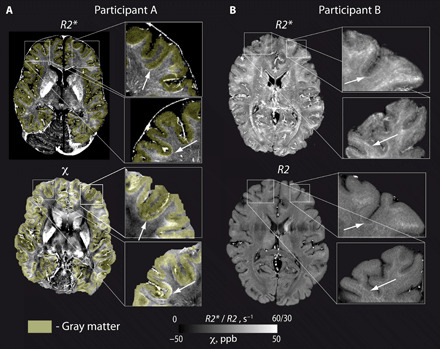
SWM is visible on QSM, R2*, and *R2* maps in vivo. (**A**) R2* (top) and QSM maps (bottom) from a representative participant show thin SWM strip (white arrows) just below the cortex with elevated R2* and magnetic susceptibility. The maps are overlaid with a cortical GM mask (yellow transparent), based on synthetic *T1*-weighted images generated from quantitative *R1* and *PD* maps. (**B**) R2* (top) and *R2* (bottom) maps obtained on another participant reveal elevated R2* and *R2* in SWM.

R2*, *R2*, and χ were strongly increased in a thin tissue strip underneath the cortex (Fig. 1). This hyperintense strip, 0.5 to 2 mm thick (as estimated at different locations in the brain), was identified as SWM due to its location below the GM-WM interface (Fig. 1A). The visibility of this strip varied across the brain areas (Fig. 1A).

**Fig. 2:**
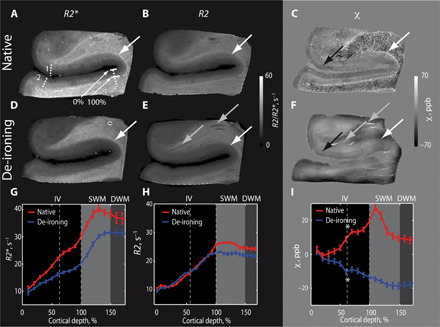
MR-contrast of SWM is eliminated by tissue iron removal. Quantitative maps of (**A** and **D**) R2*, (**B** and **E**) *R2*, and (**C** and **F**) χ of a postmortem brain sample of the temporal lobe were recorded before (A to C) and after (D to F) de-ironing. Cortical and subcortical profile curves are shown of averaged (**G**) R2*, (**H**) *R2*, and (**I**) χ. Averaging was performed over the sulcal region within the borders indicated in (A) by the dashed lines. Bars represent SEM across profiles. Before de-ironing R2*, *R2*, and χ are increased in a thin stripe underneath the cortex (large white arrows) and in cortical layer IV [black arrows in (C) and (F) and asterisks in cortical profiles in (I)]. After de-ironing the high intensity stripe in R2*, *R2*, and χ vanished, while the contrast between WM and GM remained preserved. The χ contrast in layer IV is reversed after de-ironing due to the negative susceptibility of myelin [indicated with asterisks in (I)]. Some of the small vessels changed their appearance (gray arrow)—most probably due to wash out of remaining blood from the tissue. Slow smooth intensity variations in the χ maps after de-ironing are in line with potential tissue alternation by the de-ironing procedure.

Similarly, postmortem brain tissue samples from the temporal lobe also exhibited substantially elevated R2*, *R2*, and χ values in the SWM (Fig. 2, A to C). The averaged cortical profiles of *R2*, R2*, and susceptibility peaked within the SWM (Fig. 2, G to I), distinguishing it clearly from GM and DWM. The maximum χ value in the averaged cortical profiles was found in SWM. A positive shift difference, between SWM and DWM susceptibility values was χ_SWM_ − χ_DWM_ = (21 ± 3) parts per billion (ppb) (means ± SD) (Fig. 2, C and I). The averaged R2* was significantly higher in SWM compared to DWM, whereas *R2* was only slightly increased in SWM: ΔR2* = (10 ± 0.8) s^−1^ (28% of the DWM R2* value) and Δ*R2* = (3 ± 0.25) s^−1^ (12.5% of the DWM *R2* value), respectively (Fig. 2, G to H).

In postmortem tissue, the orientation dependence of the SWM contrast was investigated by comparing R2* values recorded at two different orientations of the sample with respect to the main magnetic field (fig. S1). Although some orientation-dependent contributions to the SWM R2* were identified, the contrast between SWM and DWM did not depend on the orientation with respect to the magnetic field. The effects may be tentatively attributed to orientation-dependent contributions of myelin to the relaxation rates (*14*).

The positive susceptibility shift and increased R2* and *R2* relaxation rates in SWM, both in vivo and in postmortem brain samples, supports earlier reports suggesting paramagnetic iron as the underlying contrast driver (*17*). The higher values of ΔR2* compared to Δ*R2* are in line with a substantial contribution of static intravoxel dephasing to R2* and thus an increase of *R2*′ (= R2* − *R2*), which can be explained by mesoscopic or microscopic inhomogeneous iron distributions within the MRI voxel (*23*).

### MR contrast in SWM disappears after iron extraction

A tissue metal extraction experiment was performed to corroborate the role of iron in generating the SWM contrast and to quantify the relative impact of the local iron and myelin distributions on the MRI parameters. Parameter maps were obtained on postmortem brain samples before and after iron extraction with a deferoxamine mesylate salt solution (Fig. 2). The 0.5- to 2-mm-thin SWM strip with enhanced *R2*, R2*, and χ values, apparent before iron extraction (Fig. 2, A to C), vanished after iron extraction (Fig. 2, D to F). Furthermore, the maxima of the averaged profiles of *R2*, R2*, and χ, located within SWM, disappeared after iron extraction (Fig. 2, G to I). R2* and *R2* in SWM were reduced by R2*_before_ − R2*_after_ = (12 ± 2) s^−1^ and *R2*_before_ − *R2*_after_ = (6 ± 0.6) s^−1^, respectively (means ± SD). The vanishing contrast after iron extraction provided a direct indication that the difference in *R2*, R2*, and χ between SWM and DWM originated primarily from the elevated level of paramagnetic iron in SWM. While iron’s contribution dominates the R2* contrast between SWM and DWM, it represents a substantial, but not the dominant, part in transverse and effective transverse relaxation rates, explaining about (22 ± 2) % of the total *R2* and (32 ± 7) % of the total R2* in SWM.

Note that after iron removal, no differences between the susceptibility of SWM and DWM [χ_SWM_ − χ_DWM_ = (−2 ± 5) ppb] was measured within experimental error, indicating that the myelin density in SWM is comparable to that in DWM. The WM appeared patchy in the R2* and susceptibility maps before iron extraction but homogenous after iron extraction (fig. S2, top right). The patches of alternating hypo- and hyperintensity may be caused by patchy iron distributions in the WM (fig. S2, bottom right), as has also been observed previously (*19*–*21*).

### Quantitative histology at mesoscopic and microscopic resolutions confirms dominating contribution of iron

**Fig. 3:**
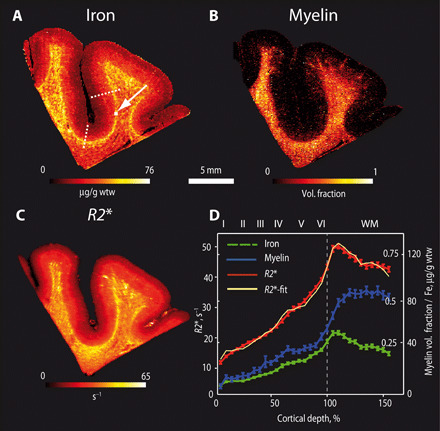
Elevated iron levels determine MR contrast in SWM. (**A**) Quantitative iron map and (**B**) estimated myelin volume fraction map were obtained with LA-ICP-MSI and compared to (**C**) quantitative R2* map. Maps depicted in (A) to (C) were obtained from the same postmortem brain sample slice of the temporal lobe. Elevated iron levels in a thin (0.5 mm) stripe in the SWM dominate the R2* contrast. Note that the myelin volume fraction is not elevated in the SWM compared to DWM. (**D**) Averaged cortical and subcortical profiles of iron, myelin, and R2*, obtained in the sulcus between the positions marked with the dotted line in (A). R2* fits, calculated based on the linear combination of myelin and iron contributions, are also shown in (D) (in yellow). Bars represent SEM across the profiles. The white square highlighted by the arrow in SWM in (A) indicates the position of the PIXE measurements shown in Fig. 4B.

To link ΔR2* and Δχ to tissue composition in SWM, the iron and myelin distributions were quantitatively mapped at the mesoscopic and microscopic scale with advanced histology methods and compared to postmortem MRI of the same tissue block. Quantitative iron distribution maps obtained by LA-ICP-MS revealed a 0.5- to 2-mm-thin strip with elevated iron levels below the WM to GM interface (Fig. 3A and fig. S3C). The iron concentration in the SWM was (55 ± 11) μg/g wet tissue weight (wtw). This value was significantly higher than in the upper cortical layers (spanning from pial surface to 25% of cortical depth) [(15.6 ± 4) μg/g wtw], middle cortical layers (spanning from 30 to 65% of cortical depth) [(29.2 ± 7) μg/g wtw], and in DWM [(33 ± 10) μg/g wtw].

In contrast to the iron maps, the myelin volume fraction maps estimated from the measured quantitative phosphorus and sulfur concentrations (Fig. 3B) and myelin basic protein stain (fig. S4B) did not show any significant enhancement in the SWM myelin density compared to DWM. There is a notable similarity between the maps and cortical profiles of iron concentration (Fig. 3A) and R2* (Fig. 3C), emphasizing that iron strongly contributes to R2* in both GM and WM.

### High iron concentration observed in SWM oligodendrocytes

**Fig. 4:**
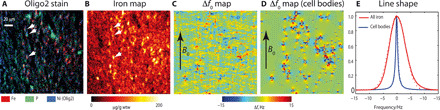
Cellular distribution of iron dominates iron-induced R2* relaxation in SWM. (**A**) Oligodendrocytes in SWM were visualized by PIXE elemental maps of phosphorus (green), iron (red), and nickel (blue, Ni-enhanced Olig2 stain for oligodendrocyte cell somata). Locations of several oligodendrocytes are marked with white arrows. (**B**) Quantitative map of iron concentration in SWM was obtained with PIXE (see Fig. 3A for the position of the 200 μm by 200 μm field of view with respect to the brain slice). (**C**) Simulated map of microscopic intravoxel Larmor frequency perturbations was calculated using the cellular iron distribution shown in (B). (**D**) Simulated map of Larmor frequency distribution resulting from the iron-rich oligodendrocyte bodies is shown. This distribution was obtained by thresholding the map shown in (B) at the level of 70 μg/g wtw. (**E**) Line shape of water MR signal resulting from the Larmor frequency distributions [(C) and (D)] was best described by Gaussian and Lorentzian line shapes, respectively. The direction of *B*_0_ is indicated by a black arrow in (C) and (D).

The iron concentration in SWM was mapped to specific cell types and subcellular compartments using PIXE with 1-μm resolution within a field of view of 200 μm by 200 μm (Fig. 4B) (similar to the voxel sizes of the postmortem MRI experiment). Hotspots of iron concentration with an extent of approximately 5 μm were localized in SWM. Comparison with immunohistochemistry revealed that these hotspots were colocalized with the somata of oligodendrocytes and some astrocytes in the SWM (Fig. 4A). We found that not all oligodendrocyte somata had the same iron content.

The oligodendrocyte bodies contained 12% of the overall iron mass in the sub-volume scanned with PIXE. The remaining 88% of iron detected outside the SWM oligodendrocyte somata might be attributed to iron in oligodendrocyte processes or myelinated fibers, since a high similarity between phosphorus (as coarse cell membrane and myelin marker), immunohistochemically marked oligodendrocytes, and iron distributions in the PIXE maps (Fig. 4A) was found. The distribution of neurons and microglia differed prominently from the observed iron distribution (fig. S4), excluding them as relevant iron-containing microstructure. However, a precise assignment of this iron to a particular cellular compartment was not possible with the available PIXE resolution.

### Empirical linear model of R2* in SWM requires separate iron and myelin contributions

To quantify the contribution of iron and myelin to R2*, we first used the empirical linear model proposed in reference (*21*).The averaged cortical profile of R2* was modeled as a linear combination of the iron and myelin concentration profiles. The best fit was provided by the following parameter set

$$R2^{*}=(0.35\pm 0.25s^{-1})c_{\text{Fe}}+(47\pm 7s^{-1})\nu_{m}+13.7s^{-1}$$ (1)

where *c*_Fe_ is the iron concentration in μg/g wtw, *v*_m_ the myelin volume fraction, and the constant term (13.7 s^−1^) is an offset describing any relaxation processes unrelated to iron and myelin variations (Fig. 3D). This model explained 91% of the variance in cortical profiles, whereas an alternative model including only the myelin contribution and a constant term explained only 66% of the variance. The strongly improved fit and high relaxivity of iron estimated by the linear model provides additional support for the important role of iron in R2* contrast generation in SWM.

### Generative model of R2* relaxation in SWM needs to account for cellular iron distribution and orientation dependence of R2*

The empirical linear model described by Eq. 1 has several limitations. First, it does not provide understanding of the microstructural underpinnings of iron-induced R2*, which is indispensable for the interpretation of R2* data. Second, it does not account for the potential dependence of R2* parameters on tissue orientation in the magnetic field (fig. S1) (*24*), which is important for whole-brain SWM mapping.

To mechanistically link the microscopic and mesoscopic iron distribution in SWM to R2*, we developed a generative biophysical model of iron-induced relaxation in SWM. This model accounts for the contribution of iron and myelin, as well as the magnetic field orientation dependence. Our complete theoretical considerations are provided in Materials and Methods. Here, we summarize the key findings relevant for SWM mapping of the entire brain. In the following, we assumed that R2* can be considered as a sum of reversible (*R2*′) and irreversible contributions (*R2*), i.e., R2* = R2 + R2′ for simplicity.

In the human brain, iron is mostly stored in paramagnetic form in the protein ferritin (*25*). Ferritin-bound iron contributes to R2* via three distinct relaxation mechanisms operating at different temporal and spatial scales. At the nanoscale from tens of angstroms to hundreds of nanometers, fast fluctuating molecular interactions of water spins with ferritin-bound iron lead to irreversible transverse relaxation *R2* (*23*, *26*). At the microscale spanning from micrometers to tens of micrometers, the heterogeneous cellular distribution of iron induces perturbation of local magnetic fields and therefore R2* relaxation (*23*). Last, at the submillimeter mesoscale of the MRI voxel size, variation of the magnetic susceptibility within the SWM strip results in intravoxel signal dephasing and therefore contributes to reversible *R2′*. Relaxation mechanisms resulting from these three mechanisms have different relative contributions to *R2′* and *R2*. Moreover, contributions of the three mechanisms depend on different aspects of the tissue iron distribution and reveal a different dependence on the orientation of the SWM surface with respect to the static magnetic field. For example, nanoscale relaxation contributes to *R2* only, is orientation-independent, and depends solely on the mean tissue concentration of iron (see Eq. M2). In contrast, microscale relaxation contributes potentially to both *R2′* and *R2* and is determined by the cellular iron distribution. Last, mesoscale iron-induced relaxation is orientation dependent and contributes only to reversible *R2′* (see Eq. M4).

Using theoretical considerations and quantitative iron maps obtained with LA-ICP MSI and PIXE, we estimated the contributions of nanoscale, microscale, and mesoscale relaxation processes to iron-induced R2* in SWM as described below. The nanoscale contribution of iron to *R2* (and therefore to R2*) was estimated using the averaged iron concentration in SWM (55 ± 11 μg/g wtw) obtained from the LA-ICP-MS experiment (Fig. 3) and Eq. M2. The resulting contribution of the nanoscale relaxation to R2* in SWM was Δ*R2*_nano_ = (1.2 ± 0.2 s^−1^). This contribution is only a small fraction of the experimentally measured iron-induced relaxation rate of (12 ± 2) s^−1^ obtained in the tissue metal extraction experiment.

The mesoscale contribution to R2* was estimated using differences in the iron concentrations of SWM and DWM, measured with LA-ICM-MSI (22 ± 21 μg/g wtw) and by applying Eq. M4. Under the assumption of a perpendicular orientation of the SWM slab to the static magnetic field (see Eq. M4), the ΔR2′_meso_ = 3.4 s^−1^ was estimated (fig. S6C).

The microscale contribution to R2* was estimated using microscopic maps of iron concentration obtained with PIXE. To estimate the line broadening, intravoxel distributions of proton resonance frequencies were predicted from cellular iron maps measured with PIXE (Fig. 4B). To investigate the influence of different cellular compartments, two simulations were performed. The first simulation estimated the effect of the entire iron content, while in the second simulation, only contributions from the iron-rich cell somata were taken into account. The resulting field maps and histograms for both cases of intravoxel Larmor frequency distributions are presented in Fig. 4, respectively.

Consideration of the total iron content led to a distribution of proton Larmor frequencies within the voxel that was well described by a Gaussian with full width at half maximum (FWHM) of 8 s^−1^ (Fig. 4E). Restricting the model to the contributions of the iron-rich cell somata resulted in a line shape close to a Lorentzian distribution with a FWHM of 1.2 s^−1^. Predictions from the first simulation model were in good agreement with experimentally obtained values from the iron extraction experiment, which revealed an impact of the tissue iron on the R2* contrast of ΔR2* = (12 ± 3) s^−1^. By comparing the simulations obtained from the two tested models with the experimental results, the influence of the iron-rich fibers can be identified as the dominant contribution, which is bigger than the effect of the iron-rich cell somata. This result clearly shows that the applied approach is not only capable of assigning iron as the main source of MR contrast in SWM but also allows the prediction of MR relaxation rates based on the microscopic iron distributions. The microscopic contribution to R2* scales linearly with the total iron concentration. By dividing the obtained linewidth of 8 s^−1^ by the total iron concentration in an SWM voxel investigated with PIXE (37 μg/g wtw), the effective microscopic relaxivity (r2*_micro_) was estimated r2*_micro_ = 0.215 s^−1^/ μg/g wtw, which compares well with the relaxivity factor (0.35 ± 0.25 s^−^/ μg/g wtw) found in the empirical model (Eq. 1). In summary, we found iron-induced contributions to ΔR2* according to ΔR2*_micro_ > Δ*R2′_meso_ > *Δ*R2*_nano_, indicating that the dominating contribution originates from the microscopic scale.

### R2* and QSM in the SWM vary across brain areas with sharp boundaries between cortical areas

**Fig. 5:**
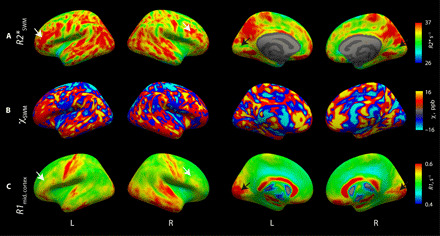
SWM contrast varies across the brain. (**A**) R2* in SWM (defined as a surface 0.5 mm below the cortical GM-WM interface) corrected for orientation dependent contributions of iron and myelinated fibers reflects iron accumulation in SWM. Low values were found in the primary visual cortex (dark arrow). High values were visible in the frontal (white arrow), temporal, and parietal association areas. (**B**) Susceptibility maps of SWM. (**C**) Intracortical *R1* maps at the middle cortical surface. High *R1* values are seen in the highly myelinated primary visual, motor, and somatosensory cortical areas. The primary visual area with high intracortical myelination (black arrow) exhibits low R2* values in the adjacent SWM, while association areas with low intracortical myelination (e.g., white arrow) show high SWM R2* values.

The iron deposits in SWM were mapped over the entire human brain in vivo using R2* maps in combination with the generative model (Eq. M8) and QSM maps. Whole-brain multiparametric data were acquired in four participants with an isotropic resolution of 400 μm, which yielded intrinsically co-aligned maps of longitudinal relaxation rate (*R1*), PD, R2*, and QSM. The high contrast between GM and WM on *R1* and PD maps was exploited to obtain the cortical GM-WM boundary. SWM was then determined as the surface 0.5 mm below the GM-WM boundary, and R2* and susceptibility values were mapped at this depth across the entire brain (Fig. 5, B and C). We defined the SWM as a surface running at a constant depth from the cortical GM-WM boundary to sample quantitative MRI parameters exclusively inside the SWM and to keep the partial volume effect with GM constant across the brain areas. The orientation-dependent influence of myelin and iron on R2* was estimated and removed using the general linear model (GLM) described in Eq. M8. Intracortical myelination and myelination of SWM were estimated from the *R1* values in the middle of the cortex (Fig. 5D) and in the SWM surface (fig. S5D), respectively [following Sereno *et al*. (*27*)] to relate R2*- and QSM-based SWM maps to cortical myelination patterns.

A strong variation of iron-induced R2* was observed in the SWM across different brain regions (Fig. 5A). Higher R2* values were found in frontal, temporal, and parietal association areas, whereas lower R2* values were observed in the primary visual and the auditory cortices (Fig. 5A). Similar patterns were observed on the QSM maps (Fig. 5B). These patterns of variation across brain regions were distinct from the variation of SWM curvature (fig. S5C), SWM myelination (fig. S5D), and the orientation-dependent term in Eq. M8 (fig. S5B) and were also visible before the correction for orientation-dependent contribution (fig. S5A). The orientation-dependent terms in the model explained only 8 ± 3% (means ± SD across four participants) of the variance in whole-brain R2* maps (fig. S5B). Correction of orientation-dependent effects reduced their impact even further (fig. S1). Thus, the patterns are most likely driven by the variation of SWM iron content.

It is known that the cortical primary motor, somatosensory, and auditory areas are heavily myelinated and myelinate early during development (*27*), which is reflected in higher intracortical *R1* values (Fig. 5C). In contrast, low R2* values were observed in the SWM underneath these brain regions (Fig. 5A). This pattern reversed for the frontal and temporal association areas: Low intracortical *R1* values were colocalized with high R2* values in the SWM. This is consistent with a low density of myelinated intracortical and ascending long range fibers but a high density of short association fibers, which myelinate late during the life span (*28*). Sharp boundaries between the primary and secondary visual areas were visible in the SWM. The primary motor and somatosensory areas showed a third type of pattern with increased values of both *R1* in the cortex and R2* in SWM.

## DISCUSSION

We have demonstrated that SWM can be reliably identified and differentiated from both cortical GM and DWM, in vivo, with high-resolution quantitative *R2*, R2*, and susceptibility mapping at 7 T. The high iron concentration in the somata of oligodendrocytes and other microstructural components in SWM were identified as the main source of MR contrast by using advanced histology and postmortem MRI. A unified biophysical model was developed to quantitatively correlate MR parameters with iron distribution at the micro- and mesoscopic level. With this model, the SWM iron deposits were mapped over the entire human brain in vivo. Iron deposits were increased in the U-fiber rich frontal, temporal, and parietal association areas, while they were reduced in areas with high intracortical myelination (including primary visual and auditory areas, but not primary sensorimotor areas). The sharp boundary between the primary and secondary visual cortex, in terms of iron accumulation identified in vivo, is in line with previous studies in which histology and postmortem quantitative iron mapping were used (*20*, *29*). These findings confirm area-dependent iron concentration in the SWM and may also reflect U-fiber density or myelination patterns of the overlying cortical areas.

Our results have several important implications. First, they demonstrate that SWM iron deposits can be mapped by quantitative MRI in vivo, which holds great promise for the study of various pathologies with impaired iron homeostasis (*30*). For example, the regional distribution of plaques in AD could be partially explained by the interregional distribution of SWM iron. It has recently been shown that both plaques and tangles in AD are more prominent in sulcal fundi (where the U-fiber density is increased) than in gyral crowns (*31*). Higher vulnerability of sulcal fundi to AD could be related to enhanced SWM iron levels in these regions.

Second, structural SWM contrasts may potentially be used for in vivo mapping of cortico-cortical U-fiber densities, thus significantly advancing future studies in local brain connectivity and studies of the human connectome. Our supposition that iron-induced contrast in the SWM is related to U-fiber density is supported by the colocalization of iron with somata of oligodendrocytes, as demonstrated here by cellularly resolved iron mapping. This colocalization may reflect the specific myelination processes in SWM, since iron is recognized as an important cofactor for myelin synthesis and oligodendrocyte proliferation (*32*). Additional support for our assumption comes from the inhomogeneous SWM contrast distribution in the brain, as this excludes nonspecific global effects and supports area-specific processes or structures. The increased SWM iron content in the frontal and temporal association areas may reflect distinct myelination processes of U-fibers in these late myelinating areas, as late myelinating oligodendrocytes have an increased metabolic demand and iron is a basic requirement for oxidative metabolism, which is crucial for lipid synthesis and normal myelination (*32*).

Detailed knowledge of the U-fiber topography in the human brain, which could be used to validate our hypothesis, is very limited. Early dissection studies reported U-fiber systems in frontal, occipital, and temporal areas (*33*), which is in excellent agreement with the high SWM contrasts observed in our study. While several studies have focused on short cortico-cortical connections in the occipital lobe, precentral and postcentral gyri, and frontal (*8*) and temporal areas in humans and primates, there has been no systematic mapping of U-fibers across the entire human brain. Recent in vivo MRI studies, based on the magnetization transfer contrast (*4*), have revealed higher U-fiber densities in the frontal and temporal lobes compared to the occipital pole. The regional SWM density distributions reported for healthy control groups in the mentioned study correspond well to the iron-induced SWM contrast observed for the four participants in our study. Although the in vivo and postmortem experiments strongly suggest that R2* maps reflect U-fiber density, additional studies are needed to corroborate this claim. For example, comparisons should be made to tracer studies and to U-fiber density measurements from emerging submillimeter resolution DWI techniques that can substantially improve the detectability of subcortical U-fibers.

An alternative explanation for the observed differences in the iron content of SWM over the brain may be regional differences in its cellular organization (*5*, *6*). It has been demonstrated that the density of interstitial neurons, their type, and morphology vary across brain regions, with the lowest density of WM neurons in the visual cortex (*5*). This may explain the sharp boundary we observed between the primary and secondary visual cortex.

Generally, our findings demonstrate that iron strongly influences the MR contrast in WM. The role of iron in laminar-specific contrast in the cortex (*20*, *21*) and subcortical nuclei (*34*) has been widely recognized. However, most MR contrast models in WM neglect the iron contribution (*13*, *18*). In addition, the relationship between the microscopic iron distribution and R2* is an important step in the quantitative understanding of iron-induced contrast in the human brain and, in particular, the understanding of the differential contributions of the different cell populations to iron-induced contrast. Quantitative microscopic iron maps as obtained in our study (Fig. 4), in combination with theoretical concepts developed for R2* contributions of microscopic magnetic perturbations (*23*), pave the way for in vivo MR iron histology. Our biophysical model predicts R2* within 30% error without any free fitting parameters, which is an advancement over previous work that included free parameters or lacked quantitative predictions. The residual difference may be addressed by including iron-induced effects on *R2* resulting from water diffusion in inhomogeneous fields induced by iron-rich cells (*23*).

Our findings may be helpful in studies of the human cortex, especially of its parcellation. The SWM strip appears to be a more prominent imaging feature on R2* and susceptibility maps than the variation of myelination in cortical layer IV, which is often directly or indirectly used for parcellation (*11*, *27*). Because SWM contrast is region-dependent, cortical parcellation methods based on intracortical myelin-sensitive *R1* contrast may benefit from adding R2* and QSM information from the SWM. In addition, MRI-based segmentation algorithms could benefit from SWM contrast to better define cortical boundaries.

A limitation of our work is that only the total iron content and not the spin state was assessed. In addition, the limited resolution of PIXE (approximately 1 μm) makes it difficult to assign iron to specific subcellular compartments. It has been shown that iron is localized in outer and inner layers of the myelin sheaths (*35*). However, the precise localization of elevated iron concentrations in SWM to a specific compartment, such as myelin sheaths, oligodendrocyte processes, or the extracellular space, requires further investigations with methods capable of iron quantification at the nanometer scale.

Here*, R2* mapping was based on the acquisition of a single slice, which limits the extractable information on the distribution of *R2* across the cortical areas. Future development of the described method toward three-dimensional (3D) intrinsically co-aligned R2/R2* acquisitions may add additional information.

The proposed model of R2* contrast mechanisms relies on the following assumptions. The mass susceptibility of iron was estimated on the basis of a value for ferritin-bound iron found in the literature. This value, however, may slightly differ from the mass susceptibility of iron in brain tissue due to presence of different iron forms, contributing to systematic error of our model. Any systematic error of the assumed brain tissue density and experimentally estimated tissue shrinkage factor would influence the mass iron concentration used in the modeling. In addition, the orientation-dependent myelin contribution was estimated assuming no preferential orientation of the U-fibers within the SWM plane. In the future, more realistic fiber orientation distributions obtained from DWI may be used to improve the model. Last, the in vivo measurements are prone to partial volume effects and image processing inaccuracies, since the strip of SWM is only approximately 0.5 mm thick. In particular, the thickness and exact location of SWM with respect to the GM-WM boundary may slightly vary.

Our study was based on four in vivo datasets and one postmortem brain only. However, since our results relate mainly to the fundamental physical mechanisms of SWM contrast, we do not expect that the model and the contrast mechanism will differ in the population and influence its generalization to the healthy population. Different neuropathologies can lead to different iron distributions, which the model may not adequately explain. These situations require further study. However, the presented biophysical model, multi-parameter acquisitions, and the corresponding image processing offer the opportunity to further study the SWM contrast in pathological and/or interindividual variations.

In summary, we have presented a novel biophysical generative model and performed the first systematic investigations of SWM iron distribution throughout the brain in vivo. Our postmortem experiments provide a mechanistic explanation of the observed contrast, linking iron, late-myelinating axons, and oligodendrocytes. Our results suggest that the maps may reflect U-fiber density throughout the brain, providing a unique window into short cortico-cortical connections. The newly developed method can be used to assess inter-regional, interindividual, and developmental differences in SWM and U-fibers in healthy and pathologically altered brains.

## MATERIALS AND METHODS

### In vivo MRI measurements

#### Whole-brain R2*, R1, PD, and QSM maps

Four healthy volunteers (28 ± 1 years) were scanned over three sessions on a 7 T MR system (Magnetom 7 T, Siemens Healthineers, Erlangen, Germany) using a 32-channel radio frequency (RF) head coil (Nova Medical Inc., Wilmington, MA, USA). The study was approved by the local ethics committee. Quantitative parameter maps, including R2*, *R1*, and PD maps, were obtained using a gradient- and RF-spoiled multi-echo 3D gradient-echo sequence with 400-μm isotropic resolution [repetition time (TR) of 31.8 ms, eight equidistant echoes acquired with alternating readout gradient polarity, first echo time (TE1) of 3.4 ms, and distance between echoes (ΔTE) of 2.6 ms], readout bandwidth (BW) of 434 Hz per pixel, and matrix size of 560/640/416 (phase/read/slice) (*11*). In each session, both PD-weighted (flip angle α = 5°) and *T1*-weighted (α = 28°) images were acquired in addition to calibration data to correct for RF transmit field nonuniformity. Parallel imaging with acceleration factor of 2 was applied in both phase-encoding (PE) directions, enabling acquisition of each volume in 32 min. Prospective motion correction (Kineticor, HI) was used to correct for both intra- and interscan motion. Due to the large size of the acquired datasets, raw data were streamed online, and images were subsequently reconstructed offline using a SENSE-based parallel imaging algorithm. Sensitivity maps were estimated from integrated *k*-space reference lines (*N* = 84, *N* = 88 lines in each PE direction). Transmit RF field mapping was performed using echo planar imaging acquisition of spin and stimulated echoes with 15 different refocusing flip angles.

Quantitative R2*, *R1*, and PD maps were created from the weighted datasets using the hMRI toolbox (http://hmri.info) and SPM12 (www.fil.ion.ucl.ac.uk/spm/) within MATLAB (MathWorks, MA). Maps created from each of the three sessions were skull-stripped, coregistered to the maps of the last session using the Optimized Automated Registration as implemented in CBS Tools in MIPAV (www.nitrc.org/projects/cbs-tools/), and then averaged across the three sessions to increase signal-to-noise ratio (SNR).

For each of the three multi-echo PD-weighted acquisitions, QSM maps were reconstructed, registered to the third scan session, and averaged across scan sessions. The QSM maps were computed from the phase information of all eight echoes of the PD-weighted acquisitions. Phase discrepancies between odd and even echoes were compensated (*36*), and echoes were averaged in an SNR-efficient manner (*37*). Unwanted background phase contributions were removed using sophisticated harmonic artifact reduction for phase data with varying spherical kernels (V-SHARP; radii range, 0.4 to 4 mm) (*38*). The background-free phase data were then scaled to yield the local magnetic field distribution, and homogeneity enabled incremental dipole inversion was carried out for field-to-susceptibility inversion. (*37*) We referenced all in vivo susceptibility maps to the average susceptibility of the brain tissue within the field of view.

#### Comparison of R2 and R2*

For one participant, quantitative *R2* and R2* maps were acquired in a separate, single session. Quantitative R2* maps were obtained using a gradient- and RF-spoiled multi-echo 3D gradient-echo sequence (four echoes, TE1 = 9.18, ΔTE = 8.15 ms, TR = 44 ms, flip angle α = 14°, and isotropic resolution 500 μm) by mono-exponential fitting of the TE-dependent signal in each voxel.

A single-slice quantitative *R2* map was acquired using a 2D spin-echo (SE) sequence [TR = 2 s, flip angle = 90°, partial Fourier (PF) acquisition with 6/8 *k*-space coverage, in-plane resolution of 500 μm, slice thickness of 600 μm, and a BW of 130 Hz/Px at multiple TE = 15, 30, 40, 50, 60, 80, 100 ms] and voxel-wise mono-exponential signal fit to the multiple echoes. The orientation parameters in the scanner space were used to coregister the *R2* and R2* maps. Visual inspection ensured satisfactory registration of the maps.

#### SWM microstructure mapping

Visual inspection of R2* and QSM maps revealed that a surface 0.5 mm below the GM/WM boundary matched well the thin hyperintense stripe in SWM. To obtain the SWM surface, a synthetic *T1*-weighted dataset with optimal GM and WM contrast was generated for each subject from the *R1* and *PD* maps using FreeSurfer’s “mri_synthesize” function. Synthetic images were denoised by applying the BM4D algorithm, a nonlocal transform-domain filter for volumetric data denoising (www.cs.tut.fi/~foi/GCF-BM3D). The denoised images were conformed to 1-mm isotropic resolution and used as input into FreeSurfer’s cortical surface reconstruction pipeline. Minor segmentation errors in the resulting cortical surface reconstruction were corrected by means of manual interventions in FreeSurfer. Last, SWM was defined as a surface located 0.5 mm below the GM/WM boundary (FreeSurfer’s “white” surface).

SWM microstructure was probed by sampling the *R1*, R2*, and QSM maps at the SWM surface. *R1* maps of each subject were further sampled at middle cortical surface (50% cortical depth using FreeSurfer’s equidistant approach) to assess the pattern of intracortical myelination. All maps were smoothed across the surface (FWHM kernel of 6 mm) and resampled to an average surface template (“fsaverage”) in FreeSurfer.

To separate orientation-dependent myelin and iron contributions to SWM R2* relaxation values, we modeled these contributions as nuisance variables in a GLM using Eq. M8.

The angles between fibers in SWM and the static field, *B*_0_, were estimated using surface normals. These orientation maps were smoothed using the same 6-mm smoothing kernel as for the quantitative maps, transformed to the fsaverage template, and used as a confound regressor. Myelin-corrected R2* maps were computed for each subject by subtracting the myelin-induced and iron-induced orientation-dependent terms β*_1_*·sin^2^θ *+* β*_2_*·sin^4^θ from the R2* map. Corrected R2* maps were then averaged across all four volunteers. The *R1* map sampled in SWM, served as a myelin proxy to control for potential variations of myelin density in SWM throughout the brain.

### Postmortem MRI measurements

A block of a human postmortem brain (male, 78 years, postmortem time before fixation 16 hours, temporal lobe, Brodmann areas 20, 21, 41, 42) was obtained from the Leipzig Brain Banking Centre of the German Brain Banking Network “BrainNet,” operated by the Paul Flechsig Institute of Brain Research (Medical Faculty, University of Leipzig, Department of Neuropathology, University Hospital Leipzig). The entire procedure of case recruitment, acquisition of the patient’s personal data, the protocols, and the informed consent forms, performing the autopsy, and handling the autopsy material has been approved by the responsible authorities (approval by the Sächsisches Bestattungsgesetz von 1994, 3. Abschnitt, §18, Ziffer 8; GZ 01GI9999-01GI0299; approval no. WF-74/16, approval no. 82-02, and approval no. 205/17-ek).

The brain block was fixed in phosphate-buffered 4% paraformaldehyde solution (pH 7.4) for 6 weeks. One week before MR scanning, the brain sample was transferred to 0.1 M phosphate-buffered solution (PBS; pH 7.4) to allow for the washout of formaldehyde from the tissue.

Two smaller subsamples were dissected from the block for further analysis. Dissection was guided by T2*-weighted MRI images of the whole block. The two subsamples showed a pronounced contrast in SWM on the R2* maps.

For MRI scanning, the samples were placed in a 60-mm-diameter acryl sphere filled with PBS. Two thin acryl foil springs secured the sample in the center of the sphere. MRI images were recorded in the same 7 T MRI scanner as used for the in vivo experiments but using a custom-built two-channel transmit/receive RF coil. High-resolution quantitative maps of *R2*, R2*, and susceptibility χ were obtained for the two smaller subsamples using the approaches described above. R2* and susceptibility maps were obtained using a gradient- and RF-spoiled multi-echo 3D gradient-echo sequence (nine echoes, TE1 = 6.7 ms, ΔTE = 8.52 ms, TR = 100 ms, BW = 190 Hz per pixel, and PF = 6/8) with 210- and 160-μm isotropic resolutions, respectively. Quantitative *R2* maps of a single slice were obtained by acquisitions of the 2D SE sequence (TR = 2 s, flip angle α = 90°, PF = 6/8, in-plane resolution of 210 μm, slice thickness of 600 μm, a BW of 104 Hz per pixel, and at multiple TE = 15, 30, 40, 50, 60, 80, 100 ms).

To study the orientation dependence of the MR parameters, one postmortem sample was imaged at two orientations with respect to the main magnetic field *B*_0_. Between the two acquisitions, the sample was rotated by 73° about an axis perpendicular to the field. QSM maps were reconstructed using the same approach as for the in vivo scans. The postmortem QSMs were referenced to the average susceptibility of the tissue sample and its embedding medium. To improve visual conspicuity, we manually segmented the brain tissue from the embedding medium and presented masked images. As the experimental setup was identical for all postmortem tissue samples, the susceptibility profiles between them could be compared. However, special care should be taken when transferring the postmortem results to the in vivo maps.

Averaged cortical profiles of quantitative MRI parameters and iron and myelin concentrations were extracted for postmortem tissue blocks. To this end, manual segmentation into GM and WM was performed on several consecutive slices. Surface normals with respect to the GM-WM boundary were generated at 20 equidistant points along the WM boundary in sulcal walls and the fundus located between two gyri (see dashed lines in Figs. 2 and 3). The surface normals were extrapolated from the cortex into the WM for the length of the cortical depth at each cortical location. Quantitative R2*, *R2*, and QSM values were sampled at 40 equidistant positions along the surface normals. Obtained profiles were averaged across the 20 profiles covering the entire cortical region between the two gyri. Correspondingly, the SD at each cortical depth was calculated across the 20 sampled profiles. The averaged SWM values were determined by averaging the cortical profile values over the band spanning from the GM-WM boundary to 20% of cortical depth into the WM (corresponding to a band about 0.5 mm thickness). DWM values were calculated by averaging values between 50 and 100% of cortical depth into the WM. Cortical layer IV was identified in immunohistochemical stains for myelin as a band of dense intracortical fibers. Location of layer IV in MRI images at each position along the sulcus was determined by manual coregistration between histology and MRI images. Averaged depth location of cortical layer IV in the cortical profiles is indicated on Figs. 2 and 3.

The same procedure of extracting a cortical profile was applied to the quantitative iron and myelin concentration maps obtained with LA-ICP-MSI. The cortical profiles of iron and myelin concentrations were used as regressors in the GLM describing the cortical profile of R2* (Eq. 1 and Fig. 3D). Two models were used: one containing both myelin and iron profiles as predictors and a reduced model containing only myelin. For both models, the variance of the residuals after regression was compared with the total variance of the original data, and the percentage of the explained variance was calculated.

### Tissue iron extraction

One of the two smaller subsamples was subjected to an iron extraction procedure (*20*, *21*) to quantify iron-induced contributions to R2*, *R2*, and χ. After MR scanning, this sample was cut into two equally sized pieces. One half of the sample was incubated in a solution of 2% deferoxamine mesylate salt (Desferal) and 2% sodium dithionite dissolved in PBS at 37 C° for a period of 15 days. The deferoxamine solution was exchanged every 3 days. The other half of the sample was incubated in pure PBS under the same conditions and served as a control. Subsequently, MR scans were obtained from both samples using the same acquisition parameters as before iron extraction.

### Histology and tissue preparation

After completing MRI, the same tissue blocks were processed for histology and quantitative iron mapping. After dehydration in increasing ethanol concentrations the samples were embedded in paraffin (Histowax). Frontal sections of 12-μm thickness were cut with a sliding microtome (SM2000R, Leica). The sections were transferred to Superfrost Plus glass slides, deparaffinized with xylene, rehydrated in decreasing concentrations of ethanol, and transferred into 0.01 M PBS. Consecutive slices were immunohistochemically and histochemically stained for bright-field microscopy and PIXE analysis or left unstained for LA-ICP-MSI.

Staining was performed to analyze the distribution of potentially iron rich cell types and the distribution of myelin, iron, and iron-proteins (transferrin and ferritin) in SWM. Neurons, oligodendrocytes, astroglia, microglia, and myelin basic protein, ferritin, and transferrin were stained using the antibodies listed in Table 1.

**Table 1:** Primary and secondary antibodies used for staining of specific compartments.

**Stained** **compartment**	**Antibodies/** **binding proteins**	**Source**	**Dilution**	**Treatment for antigen** **retrieval**
**Cell stains**				
Neurons (HuCD)	Mouse anti-HuCD	Molecular Probes	1:400	tris buffer, 20 min, pH 8, 90°C
Oligodendrocytes (Olig2)	Rabbit anti-Olig2	Immuno-Biological Laboratories	1:100	Citrate buffer, 20 min, pH 6, 90°C
Microglia [(Iba 1) ionized calcium binding adapter molecule 1]	Rabbit anti-Iba1	Wako	1:800	/
Astroglia [(GFAP) glial fibrillary acidic protein]	Rabbit anti-GFAP	Dako	1:500	/
Astroglia [(GLT-1) glial glutamate transporter 1]	Guinea pig anti-GLT-1	Millipore	1:500	/
**Myelin stains**				
Myelin basic protein (MBP)	Rat anti-MBP	Abcam	1:400	
Myelin oligodendrocytes (CNPase)	Mouse anti-CNPase	BioLegend	1:300	Citrate buffer 20 min, pH 6, 90°C
**Iron-proteins**				
Ferritin	Goat anti-Ferritin	Santa Cruz Biotechnology	1:200	/
Transferrin	Rabbit anti-Transferrin	Abcam	1:5000	/

Before staining, the slices were treated for 1 hour with 60% methanol and 2% H_2_O_2_, followed by 1-hour incubation in a blocking solution (2% bovine serum albumin, 0.3% milk powder, and 0.5% donkey serum) to reduce unspecific staining. Primary antibodies were incubated in blocking solution overnight at 4°C. After incubation, brain slices were washed in PBS-Tween (0.02% Tween 20, pH 7.4) three times and then incubated with secondary biotinylated antibodies (1:1000; Dianova) for 1 hour at room temperature. All brain slices were three times washed in PBS-Tween followed by 1-hour incubation with peroxidase-conjugated avidin (ExtrAvidin, Sigma-Aldrich; 1:2000) at room temperature and rinsing in tris-HCl (pH 8.0). The staining was enhanced by 3,3′-diaminobenzidine (Sigma-Aldrich) and nickel (nickel ammonium sulfate, purity grade of 99.999%; Sigma-Aldrich) in tris-HCl (pH 8). Brain slices were lastly rinsed in tris-HCl and PBS-Tween again. In addition, Perls’ (Fe^3+^) and Turnbull’s (Fe^2+^) staining for the two chemical forms of iron were performed.

For quantitative elemental imaging with LA-ICP-MS, the unstained brain sections were dehydrated in increasing ethanol concentrations and air-dried. For quantitative PIXE, the immunohistochemically stained brain sections, still on Superfrost Plus object slides, were embedded in a mounting medium (DePeX, Merck) and subsequently removed from the object slides. The 16-μm-thick DePeX foils containing the brain sections were placed into aluminum frames for PIXE analysis.

### Quantitative iron microscopy with PIXE

Quantitative elemental maps with microscopic resolution were obtained by PIXE using the high-energy ion nanoprobe LIPSION at the Leipzig University. LIPSION provides a 1-μm proton beam with an energy of 2.25 MeV. The proton beam was scanned over multiple 200-μm by 200-μm sized brain regions, and the induced x-rays emitted from the sample were recorded. A total charge of about 70 μC was accumulated for each sample. For iron quantification, Rutherford backscattering (RBS) spectra were used to calibrate for particle exposure. RBS is used to measure the energy of proton backscatter from the sample, thereby allowing to determine the organic composition (carbon, nitrogen, and oxygen and that of hydrogen indirectly) of the sample, and thus permitting the simultaneous detection of both low and high atomic number (Z) elements when combined with PIXE. From the recorded x-rays, tagged with the position, quantitative element maps were created using dynamic analysis, which is part of the GeoPIXE II software (http://nmp.csiro.au/GeoPIXE.html). The maps were smoothed with a Gaussian filter with 2-μm kernel. Quantitative iron, phosphorus, sulfur, and nickel maps were obtained as described in (*21*). Quantitative volume iron concentrations obtained with PIXE and LA-ICP-MSI on tissue sections were converted into mass iron concentrations using a density of the brain tissue of 1.05 g/ml and an experimentally determined tissue volume shrinkage factor of 0.7. This value was obtained by a comparison of the distances between landmarks identified in optical microscopy and MRI images of the studied samples. The total iron concentration in the SWM tissue was calculated by integrating the iron content over the investigated regions. In addition, the iron fraction contained in the oligodendrocyte bodies within the studied regions was calculated using oligodendrocyte body masks. The latter were manually segmented on the PIXE nickel maps. On the maps, they were made visible by nickel enhanced immunohistochemical staining.

### Quantitative iron mapping with LA-ICP-MSI

For LA-ICP-MS iron mapping, tissue sections were ablated continuously in line-by-line scans using a commercial laser ablation (LA) system (NWR213, ESI, Portland, USA), operating at a wavelength of 213 nm with a laser spot diameter of 150 μm, energy fluence of 0.06 J/cm^2^, scan speed of 120 μm/s, and a repetition rate of 20 Hz. To ensure full sample removal, overlapping laser spot scans (30 μm overlap) were applied during line scanning.

The ablated tissue was transported with a helium gas flow of 1 liter/min to an ICP sector field mass spectrometer (Element XR, Thermo Fisher Scientific, Germany). Ablated tissue was ionized by an RF plasma source with a power of 1350 W using argon as plasma gas and auxiliary and transport gas, with flows of 15, 1, and 0.6 liter/min, respectively. Mass spectra were continuously recorded with mass resolution of 300 m/Δm and time averaged to a sampling time of 0.52 s. This setup provided elemental maps with a resolution of 120 μm by 61 μm.

The isotopes ^31^P, ^34^S, and ^57^Fe were selected for analysis. The less abundant isotopes of ^57^Fe and ^34^S with natural abundances of 2.2 and 4.21%, respectively, were selected for iron and sulfur concentration mapping due to strong interference from ^16^O_2_ and ^40^Ar^16^O with the most abundant isotopes ^32^S and ^56^Fe, respectively. For matrix-matched calibration of P, S, and Fe, solution drops of KH_2_PO_4_, CuSO_4_, and Fe-standard [1000 parts per million (ppm) in diluted HNO_3_] were dropped in multiple replicates onto de-ironed brain tissue sections and air-dried for calibration with a matrix-matched sample as described in (*39*).

Measured element intensity time profiles of the ICP-MS were converted to 2D maps and further processed with MATLAB. For quantification, element concentrations were integrated over calibrating spot areas of the standard drops, and calibration coefficients between ICP-MS signal intensity and element concentrations were obtained using linear regression. These coefficients were used to convert ICP-MS signal intensities of the measured isotopes into quantitative elemental maps.

Myelin volume fraction was estimated from quantitative maps of sulfur and phosphorus concentrations using the method reported by Stüber *et al*. in (*21*) (see “Myelin” maps section) and described in detail in section S5.

### Generative model of iron-induced R2* relaxation in SWM

We developed a generative biophysical model of iron-induced relaxation in SWM. On the basis of an empirical linear model (Eq. 1) and following previous work (*21*), we partitioned R2* in SWM into myelin and iron contributions

$$R2^{*}=R2_{\text{myelin}}^{*}+R2_{\text{iron}}^{*}+R2_{\text{other}}^{*}$$ (M1)

Subscripts indicate the contributions of myelin, iron, and other tissue components. We assume that the last term in Eq. M1 originates from tissue components other than myelin and iron and that it is constant across brain regions and orientation independent. In the following, we treat this contribution as a constant offset.

Paramagnetic iron in the brain, which we assumed to be stored mostly in the protein ferritin, contributes to R2*_Fe_ via three distinct relaxation mechanisms operating at different temporal and spatial scales: (i) nanoscale, (ii) microscale, and (iii) mesoscale. We also assume that R2* can be considered as a sum of reversible (*R2′*) and irreversible contributions (*R2*), i.e., R2* = R2 + R2′. This simplification is valid for fast and slow processes (*23*). In the following, we first provide theoretical considerations for each of the relaxation mechanisms separately. We then reformulate Eq. M1 according to these considerations and formulate a GLM capable of mapping iron-induced R2* in SWM across the cortical areas.

#### Nanoscale mechanism

At the nanoscale, water molecules engage in rapidly fluctuating interactions with iron-storage molecules, mainly the iron-rich protein ferritin (*23*, *26*). These interactions contribute to R2* via changes in *R2* only, since fast diffusion averaging occurs at these short time scales and distances (*23*). The nanoscale contribution to the R2* and *R2* relaxation rate constants can therefore be approximated by

$$\Delta R2_{\text{nano}}=r2_{\text{nano}}c_{\text{Fe}}$$ (M2)

where *r2*_nano_ is the relaxivity of ferritin-bound iron and *c*_Fe_ the tissue iron concentration. The relaxivity of ferritin-bound iron was measured at 7 T in ferritin solutions with physiological pH and temperature and reported as *r2*_nano_ = 0.0225 s^−1^/ppm wtw (*26*).

#### Microscale mechanism

At the microscale with a typical range between one to tens of micrometers, the paramagnetic iron distribution in cells (Fig. 4) induces local magnetic field inhomogeneities within an MRI voxel. These inhomogeneities result in static line broadening and, therefore, in a R2* decay (*23*).

PIXE iron maps were used to calculate intravoxel distributions of proton Larmor frequencies. The 3D maps of iron concentration were obtained by concatenating quantitative PIXE iron measurements performed on three consecutive slices, which were repeated three times to provide circular boundary conditions. The 3D iron maps were converted to magnetic susceptibility distributions by multiplying them with the mass magnetic susceptibility of ferritin-bound iron χ = 1.37·10^−9^ × *c*_Fe_ (where *c*_Fe_ is the tissue iron concentration in μg/g wtw) (*25*). The mass susceptibility of ferritin-bound iron is 1.37·10^−9^ per μg/g wtw (*25*). This value constitutes the upper bound of the reported mass susceptibilities of iron in the brain, which range from 1.37·10^−9^ per μg/g wtw (*25*, *40*) to 0.97·10^−9^ per μg/g wtw (*41*) and 0.8·10^−9^ per μg/g wtw (*42*), and have been determined by in vivo and postmortem QSM in combination with tissue iron quantification. Our models of the microscopic and mesoscopic contributions of R2*, therefore, may have a systematic error of about 40% due to the imprecision in the reference value.

The simulated magnetic field maps were estimated from the 3D magnetic susceptibility maps by the dipole field convolution method in Fourier space (*43*). When carrying out simulations for different orientations of the magnetic field with respect to the voxel, only 15% changes of the resulting linewidth values were observed. Consequently, we approximated the microscopic iron contribution to R2* to be orientation independent.

Two simulations were performed to quantify the influence of iron in different cellular compartments. In the first simulation, the effect of the entire measured iron was taken into account, while in the second only the contribution of the iron-rich cell somata was considered. The second magnetic field simulations were performed on the basis of iron maps thresholded at a level of 70 μg/g wtw.

Water diffusion was neglected in the theoretical considerations and a static dephasing limit was assumed to estimate the microscopic R2* contributions (*23*). Water diffusion in locally inhomogeneous magnetic fields partially averages the static line broadening. This effect reduces the overall R2* from microscopic mechanisms but enhances the contribution to *R2*. Therefore, our estimation provides an upper limit for the iron-induced microscopic relaxation rates.

#### Mesoscale mechanism

At the mesoscale, ranging from hundreds of microns to the voxel size, SWM can be considered as a tissue slab with enhanced iron concentration and therefore high magnetic susceptibility (Fig. 2C). Water protons within the continuous endless slab experience a frequency offset, which depends on the orientation of the slab with respect to the magnetic field

$$\delta \Omega =\gamma B_{0}\chi \Delta c_{\text{Fe}}({\text{sin}}^{2}\theta -\frac{2}{3})$$ (M3)

where θ is the angle between the slab surface normal and the external magnetic field *B*_0_, ∆*c*_Fe_ is the difference in iron concentrations between the SWM slab and the surrounding tissue, and χ is the volume susceptibility of ferritin-bound iron.

If the voxel size is comparable or larger than the slab thickness, then the frequency offsets within the slab will result in dephasing, which manifests itself as an orientation-dependent *R2′* contribution (see section S1 for more details)

$$\begin{array}{c} \Delta R2_{\text{meso}}^{\prime }\approx \frac{p(1-p)}{2}\delta \Omega^{2}T_{E}=\frac{p(1-p)}{2}\\ T_{E}{\left(\gamma B_{0}\chi \Delta c_{\text{Fe}}\right)}^{2}{\left({\text{sin}}^{2}\theta -\frac{2}{3}\right)}^{2} \end{array}$$ (M4)

where *p* describes the partial volume within the voxel and *T*_E_ is an echo time.

#### Myelin

The myelin contribution to R2* depends on the fiber orientation with respect to the static magnetic field. Empirically, the myelin contribution, R2*_myelin_ to ΔR2*, can be described as a sum of orientation-dependent and orientation-independent terms (*14*, *24*, *44*)

$$\Delta R2_{\text{myelin}}^{*}=C_{R2*}+a_{1}{\text{sin}}^{2}\theta^{*}+a_{2}{\text{sin}}^{4}\theta^{*}$$ (M5)

where C_R2_* is the orientation independent myelin contribution to R2*, θ* is an angle between fibers and the magnetic field, and *a*_1_ and *a*_2_ are empirical coefficients that scale orientation-dependent terms. The hollow cylinder model (*13*, *14*) provided theoretical justification of Eq. M5, demonstrating that parameters *a*_1_ and *a*_2_ are determined by the properties of the fibers, including fiber volume fraction, the *g*-ratio, and the fiber orientation dispersion [see equation 7 and Appendix A in (*14*)].

To apply Eq. M5 to SWM, we assume for simplicity that there is no preferential orientation of the fibers within the SWM slab. We therefore averaged the orientation-dependent terms sin^2^θ* and sin^4^θ* in Eq. M5, over all possible orientations of the fibers within the SWM plane. As shown in section S2, the averaged sin^2^θ* and sin^4^θ* terms can be expressed as a linear combination of sin^2^θ and sin^4^θ terms, where θ is the angle between the SWM surface normal and the static magnetic field. Therefore, Eq. M5 can be rewritten as

$$\Delta R2_{\text{myelin}}^{*}=C_{R2*}^{*}+a_{1}^{*}{\text{sin}}^{2}\theta +a_{2}^{*}{\text{sin}}^{4}\theta$$ (M6)

### Generative linear model for whole-brain SWM mapping

By inserting the total contributions of iron and myelin (Eqs. M2, M4, and M6) to the relaxation rate R2* into Eq. M1, it follows

R2SWM*=(r2nano+r2micro*)cFe+p(1−p)2TE(γB0χΔcFe)2(sin2θ−23)2+…+CR2**+a1*sin2θ+a2*sin4θ+R2others* (M7)

The terms in Eq. M7 can be divided into three different types. The first type includes orientation-independent terms that are linearly dependent on the iron concentration in SWM and can vary between different brain regions. The second type of terms contains orientation-dependent contributions of iron and myelin. The third type represents the orientation independent contributions of myelin and other tissue components. The latter contributions can be considered constant, assuming that there is no systematic variation of the myelin density in the SWM over the brain. Based on this approach, we formulated a GLM, which we used to map iron deposits in the SWM across the brain

$$R2_{\text{SWM}}^{*}=\beta_{0}+\beta_{1}{\text{sin}}^{2}\theta +\beta_{2}{\text{sin}}^{4}\theta +\varepsilon (c_{\text{Fe}})$$ (M8)

In Eq. M8, the terms $\beta_{0}+\varepsilon (c_{\text{Fe}})=(r2_{\text{nano}}+r2_{\text{micro}}^{*})c_{\text{Fe}}+\frac{2p(1-p)}{9}T_{E}{\left(\gamma B_{0}\chi \Delta c_{\text{Fe}}\right)}^{2}+C_{R2*}^{*}+R2_{\text{others}}^{*}$ is the contribution of myelin, iron, and other tissue components, which are independent of the orientation to the static magnetic field. β*_0_* term represents the averaged value across brain areas, and ε(*c*_Fe_) explains variation between brain areas. Assuming the contributions of myelin C*_R2*_ and other tissue components, R2*_others_ is constant over the brain. Then, the variation of iron content *c*_Fe_ across the brain is the main source of variance for the R2* between brain areas after regressing out orientation dependent part. Note that since terms sin^2^θ and sin^4^θ are strongly correlated these two terms could not be reliably separated from each other in GLM analysis. Since they describe effects of no interest in the SWM mapping, the colinearity did not affect the SWM mapping approach.

## Supplementary Material

aaz9281_SM.pdf

## SUPPLEMENTARY MATERIALS

Supplementary material for this article is available at http://advances.sciencemag.org/cgi/content/full/6/41/eaaz9281/DC1

## Appendix

View/request a protocol for this paper from *Bio-protocol*.
