# Supplementary material for: Superficial white matter imaging: Contrast mechanisms and whole-brain in vivo mapping
Source: Sci Adv. 2020 Oct 7;6(41):eaaz9281. doi: 10.1126/sciadv.aaz9281 (PMC7541072; doi:10.1126/sciadv.aaz9281)
Supplement: aaz9281_SM.pdf [file aaz9281_SM.pdf]

Science Advances
AAAS

advances.sciencemag.org/cgi/content/full/6/41/eaaz9281/DC1

Supplementary Materials for

Superficial white matter imaging: Contrast mechanisms and whole-brain in vivo mapping

Evgeniya Kirilina*, Saskia Helbling, Markus Morawski, Kerrin Pine, Katja Reimann, Steffen Jankuhn, Juliane Dinse, Andreas Deistung, Jürgen R. Reichenbach, Robert Trampel, Stefan Geyer, Larissa Müller, Norbert Jakubowski, Thomas Arendt, Pierre-Louis Bazin, Nikolaus Weiskopf

*Corresponding author. Email: kirilina@cbs.mpg.de

Published 7 October 2020, *Sci. Adv.* **6**, eaaz9281 (2020)
DOI: 10.1126/sciadv.aaz9281

The PDF file includes:

Figs. S1 to S6
Sections S1 to S5

Supplementary Materials

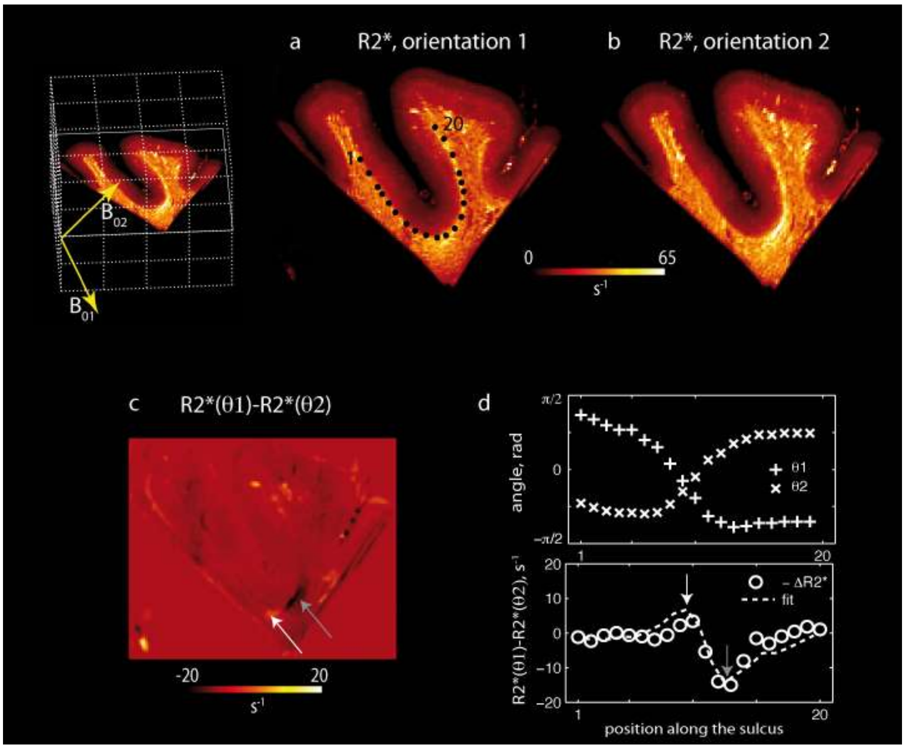

Fig. S1. Orientation-dependent contribution to *R2 in SWM.** (a, b) Quantitative *R2** measured at two different orientations of the sample with respect to the static magnetic field B0 (shown in the insert). (c) The difference of *R2** between the two different orientations shows the orientation-dependent contribution to *R2**. (d) Orientation-dependent contribution to *R2** in SWM sampled along the sulcus. Top: The orientation of SWM surface with respect to magnetic field for all sampled positions and for the two orientations of the sample in the magnetic field (θ1 and θ2 are angles between the surface normal and the static magnetic field for two sample orientations). Bottom: Orientation-dependent contribution to *R2** in SWM measured as the difference between the two sample orientations sampled along the sulcus. The sampled positions from 1 to 20 are marked in (a) with black dots. The orientation-dependent term β_1 ($\sin^2\theta 1 - \sin^2\theta 2$) + β_2 ($\sin^4\theta 1 - \sin^4\theta 2$) describing the mesoscopic contribution of iron and orientation-dependent contribution of myelin (Eq. M4, M6, S1.5 and S2.5) fitted the difference in *R2** between two sample orientations very well explaining 78% of the variance in the orientation-dependent *R2** contribution.

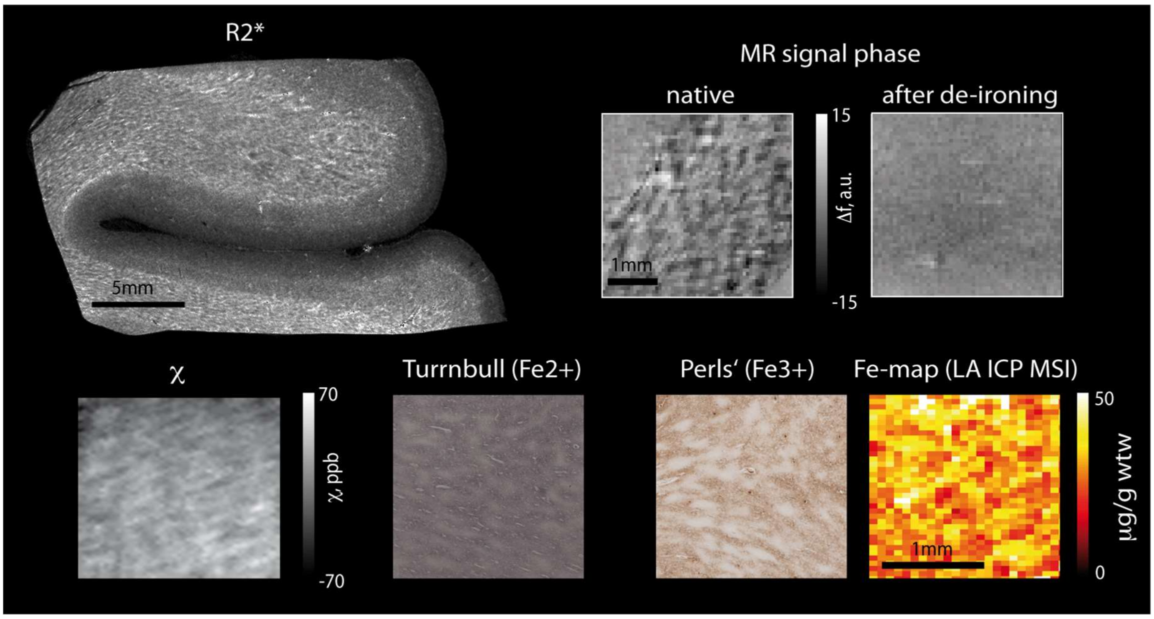

Fig. S2. Patchy appearance of deep white matter due to patchy iron distribution. Iron in WM appears in patches with characteristic sizes of 100-200 μm. The patches are apparent in classical Perls' and Turnbull's stains as well as in quantitative iron maps obtained with LA-ICP-MSI. In MRI these patches of varying magnetic susceptibility induce spatial variations of the MRI signal phase and *R2**.

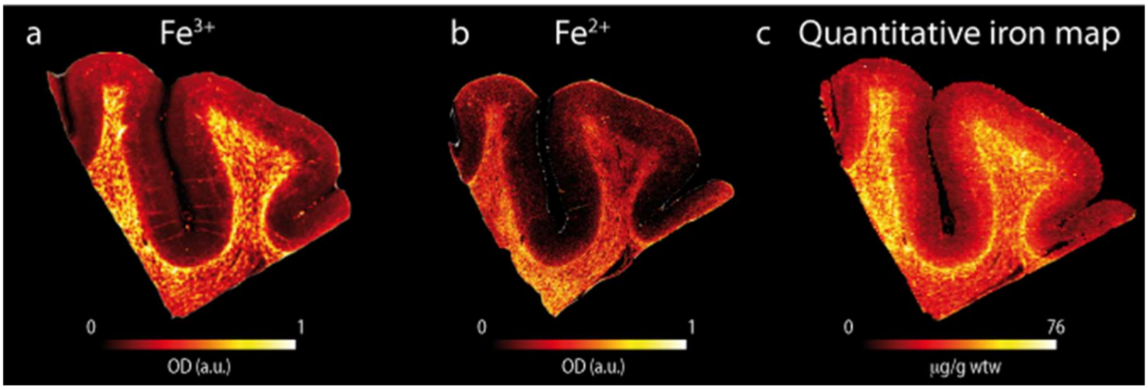

Fig. S3. Classical histological iron stains for (a) ferric and (b) ferrous iron, and (c) a quantitative iron map obtained with LA-ICP-MSI in a *post mortem* brain tissue sample of the temporal lobe. Optical images of (a) Perls' and (b) Turnbull's stainings were converted into relative maps of iron concentrations of Fe^{3+} and Fe^{2+}, respectively, using the Beer-Lambert law. The Fe^{3+} map revealed better correspondence to the quantitative iron map indicating that the majority of iron in the SWM was in the Fe^{3+} state. Note that both histological methods underestimate the cortical iron concentration.

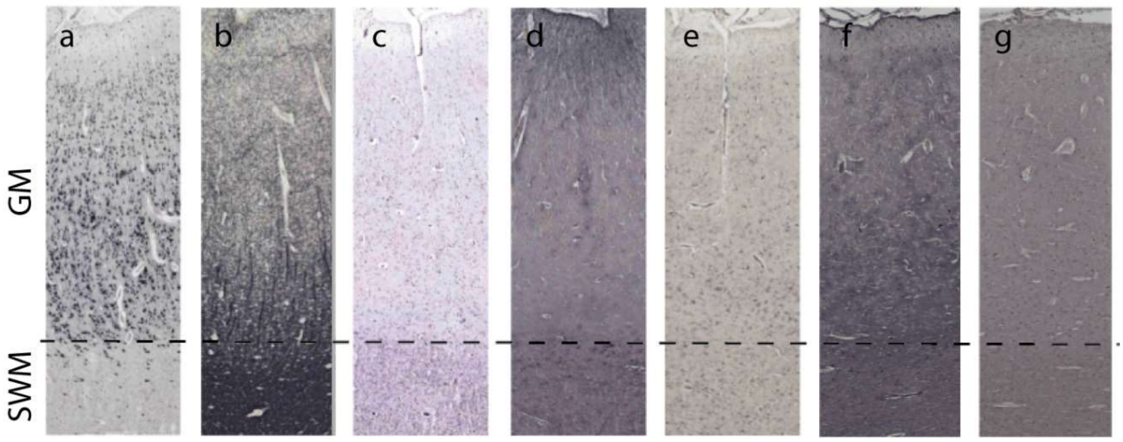

Fig. S4. Immunohistochemical stains of cortex and SWM in the sulcus for (a) neurons (HuCD), (b) myelin (MBP), (c) oligodendrocytes (Olig2), (d) astroglia (GFAP), (e) microglia (Iba 1), (f) transferrin, and (g) ferritin. Elevated densities of oligodendrocytes (c), activated astroglia (d) and transferrin (f) were detected in SWM, while microglia was homogeneously distributed across the cortex, SWM and DWM.

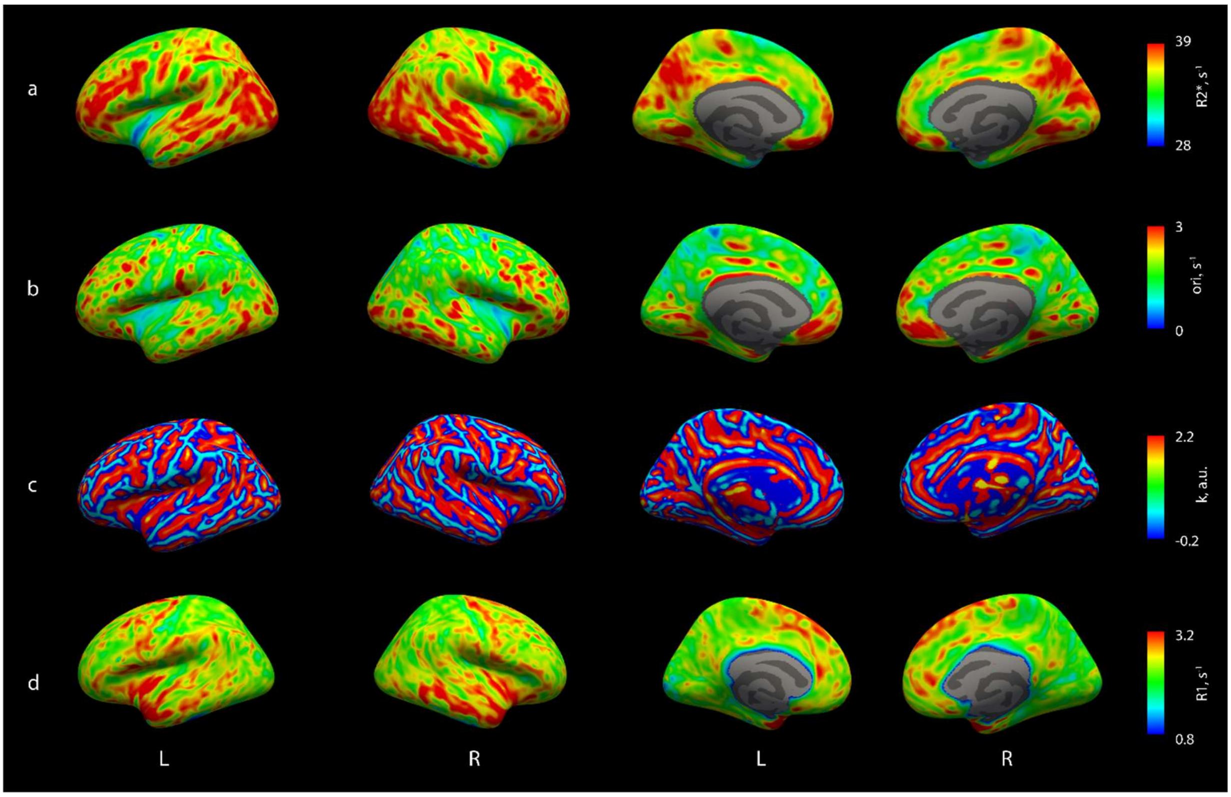

Fig. S5. Variation of geometry related metrics and *R1* in SWM. (a) *R2** in SWM defined as a surface 0.5 mm below the cortical GM-WM interface not corrected for orientation dependent contributions of iron and myelinated fibers; (b) Orientation-dependent term $\beta_1\sin^2\theta+\beta_2\sin^4\theta$ describing the orientation-dependent contribution to *R2** averaged across four participants. The orientation dependent contribution is smaller than variation of *R2** due to the accumulated iron. (c) Curvature of the WM/GM surface; (d) *R1*

sampled at the SWM surface demonstrates low variation of myelin density in SWM and patterns distinct from iron-induced SWM contrast.

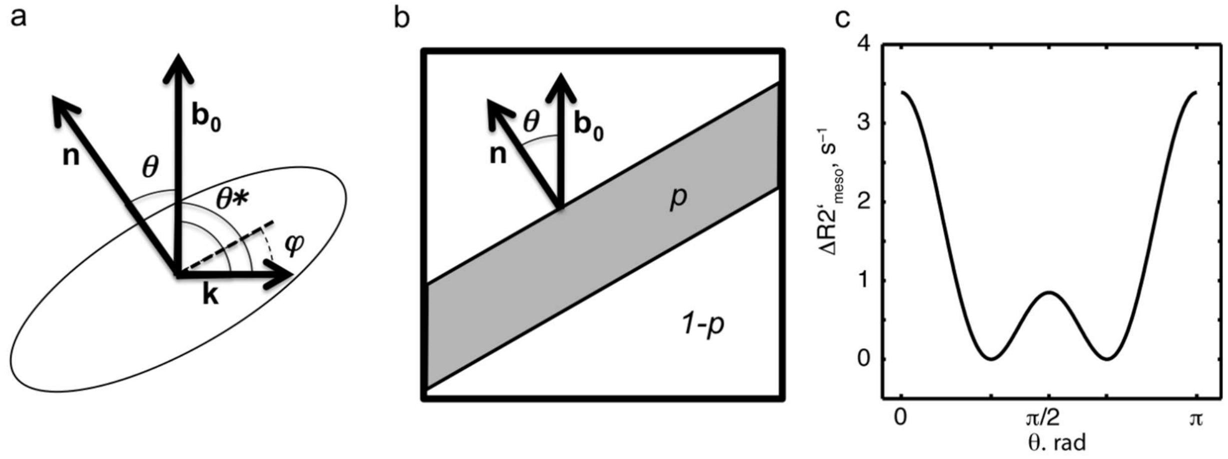

Fig. S6. Estimating orientation-dependent contribution to *R2 in the SWM.** (a) Schematic representation of SWM surface. The direction of static magnetic field $\mathbf{b_0}$ has the angle θ with the surface normal **n** and the angle θ^* with the direction of fiber bundle **k.** The in-plane angle between fiber bundle **k** and projection of the static magnetic field into the SWM plane is φ. (b) Schematic representation of an MRI voxel containing a slab of the SWM (in gray) occupying a fraction p of the voxel volume. The angle between the SWM surface normal **n** and the static magnetic field $\mathbf{b_0}$ is θ. (c) The angular dependence of the mesoscopic contribution $\Delta R2'_{meso}$ simulated using Eq. S1.5 for p=0.5, B_0=7T, $\chi=1.3\ 10^{-9}$µg/g wtw, and the differences in the iron concentrations of SWM and DWM, measured with LA-ICM-MSI (22 ± 21 µg/g wtw).

SI.1 Iron-induced mesoscopic orientation-dependent contribution to R2*

Assume that the SWM slab fills a fraction p of the voxel volume, the angle between the SWM surface normal and the static magnetic field is θ (Fig. S6b) and the difference in the iron concentration between the SWM slab and surrounding tissue is Δc_{Fe}. The frequency shift between the SWM slab and surrounding tissue is given by:

$$\delta\Omega = \gamma B_0 \chi \Delta c_{Fe} \left(\sin^2\theta - \frac{2}{3}\right) \qquad \text{Eq. S1.1}$$

The gradient echo signal from the voxel will be proportional to a sum of the signal from the SWM slab and surrounding tissue:

$$S \sim p e^{-i\delta\Omega T_E} + (1-p) \qquad \text{Eq. S1.2}$$

, where T_E is the echo time. The signal magnitude is then given by:

$$|S| \sim \sqrt{(p e^{-i\delta\Omega T_E} + 1 - p)(p e^{i\delta\Omega T_E} + 1 - p)} = \sqrt{1 + 2p(1-p)(\cos\delta\Omega T_E - 1)} \qquad \text{Eq. S1.3}$$

In our case, where $\delta\Omega T_E$<<1,[1] the Taylor expansion can be used:

$$|S| \sim \sqrt{(pe^{-i\delta\Omega T_E} + 1 - p)(pe^{i\delta\Omega T_E} + 1 - p)} \approx \cdots$$

$$\approx 1 - \frac{p(1-p)}{2}(\delta\Omega T_E)^2 \approx e^{-\frac{p(1-p)}{2}(\delta\Omega T_E)^2} \approx \qquad \text{Eq. S1.4}$$

Merging Eq. S1.4 and Eq. S1.1 the mesoscopic contribution to $R2^*$, can be written as:

$$\Delta R2'_{\text{meso}} \approx \frac{p(1-p)}{2}\delta\Omega^2 T_E = \frac{p(1-p)}{2} T_E (\gamma B_0 \chi \Delta c_{Fe})^2 \left(\sin^2\theta - \frac{2}{3}\right)^2$$

Eq. S1.5

The angular dependence of $\Delta R2'_{\text{meso}}$ is plotted on Fig. S6c, assuming p=0.5, B_0=7T, $\chi=1.37\ 10^{-9}$μg/g wtw, and the differences in the iron concentrations of SWM and DWM, measured with LA-ICM-MSI (22 ± 21 μg/g wtw,). Note that the orientation-dependent part of $\Delta R2'_{\text{meso}}$ consists of a linear combination of $\sin^2\theta$ and $\sin^4\theta$ terms.

SI.2 Myelin-induced orientation-dependent contribution to R2*

The myelin contribution to $\Delta R2^*_{\text{myelin}}$ can be described as a sum of orientation-dependent and orientation-independent terms (*24*):

$$\Delta R2^*_{myelin} = C_{R2*} + a_1 \sin^2\theta^* + a_2 \sin^4\theta^* \qquad \text{Eq. S2.1}$$

where C_{R2*} is the orientation independent myelin contribution to $R2^*$, θ^* is the angle between a fiber bundle (Fig. S6a) and the static magnetic field and a_1 and a_2 are empirical coefficients.

Since most fibers in the SWM run parallel to the SWM surface (*9*), only the contribution of fibers pointing along the SMW surface are considered in the following. For these fibers running within the SWM plane the terms $\sin^2\theta^*$and $\sin^4\theta^*$ can be rewritten as:

$$\sin^2\theta^* = 1 - \cos^2\theta^* = 1 - \sin^2\theta\cos^2\varphi$$

$$\sin^4\theta^* = (1 - \sin^2\theta\cos^2\varphi)^2 = 1 - 2\sin^2\theta\cos^2\varphi + \sin^4\theta\cos^4\varphi$$

Eq. S2.2

where θ is the angle between the SWM surface normal and the static magnetic field, and the angle between the fiber and projection of magnetic field onto the SWM plane is φ (Fig. S6a). For simplicity we assume that fibers are distributed isotropically with no preferential orientation within the plane. Performing the averaging over all fiber orientations within the plane is equivalent to integration over the angle φ. If the signal de-phasing due to the orientation dependent part of $\Delta R2^*_{\text{myelin}}$ is small[2], the averaging over φ can be performed directly on the terms in Eq. S2.1:

$$\langle e^{-(a_1\sin^2\theta^* + a_2\sin^4\theta^*)T_E}\rangle_\varphi \approx e^{-\langle a_1\sin^2\theta^* + a_2\sin^4\theta^*\rangle_\varphi T_E} \qquad \text{Eq. S2.3}$$

[1] Using differences in the iron concentrations of SWM and DWM, measured with LA-ICM-MSI (22 ± 21 μg/g wtw), magnetic field of 7T, and mass susceptibility of iron of 1.37 10^{-9} (μg/g wtw)$^{-1}$, and T_E=0.021 s the product $\delta\Omega T_E$ is estimated to be $2/3\gamma B_0\chi\Delta c_{Fe}T_E$=0.13, which is significantly less than 1.

[2] The approximation in Eq. S1.3 is justified if $(a_1+a_2)T_E$<<1. Estimating the orientation-dependent part of the $R2^*$ to be about 12 s^{-1} (as measured in the post mortem tissue Fig. S1) and multiplying it with the maximum T_E value of 0.021 s (as used in vivo experiment) the product could be estimated to be about $(a_1+a_2)T_E$ =12 s^{-1}*0.021 s=0.168, which is significantly less than 1, so the approximation is valid.

Taking into account that

$$\langle \sin^2\theta^* \rangle_\varphi = \langle 1 - \sin^2\theta \cos^2\varphi \rangle_\varphi = 1 - \frac{1}{2}\sin^2\theta$$

$$\langle \sin^4\theta^* \rangle_\varphi = \langle 1 - 2\sin^2\theta\cos^2\varphi + \sin^4\theta\cos^4\varphi \rangle_\varphi = 1 - \sin^2\theta + \frac{3}{8}\sin^4\theta$$

Eq. S2.4

Eq. S2.1 can be rewritten as:

$$\Delta R2^*_{myelin} = C_{R2*} + a_1\left(1 - \frac{1}{2}\sin^2\theta\right) + a_2\left(1 - \sin^2\theta + \frac{3}{8}\sin^4\theta\right) = \cdots$$

$$= C^*_{R2*} + a^*_1\sin^2\theta + a^*_2\sin^4\theta$$

Eq. S2.5

, where $C^*_{R2*} = C_{R2*} + a_1 + a_2$, $a_1^* = -a_1/2 - a_2$, and $a_2^* = 3/8\ a_2$. Note that the orientation-dependent part of $\Delta R2^*_{\mathrm{myelin}}$ could be described as a linear combination of $\sin^2\theta$ and $\sin^4\theta$ terms.

SI.3 Physiology, chemical form and cellular distribution of iron accumulation in SWM

Additional insight into the physiology of iron accumulation in the SWM is provided by histological stains for the different iron forms, potentially iron-rich cells, and for proteins involved in iron metabolism (Figs. S3 and S4). Classical Perls' and Turnbull's histochemical iron stains, which are specifically sensitive to Fe^{3+} and Fe^{2+} forms, respectively, revealed elevated levels of iron in both oxidation states in the SWM (Fig. S3). Perls' staining revealed higher spatial correlation with overall iron concentrations measured by LA-ICP-MS compared to Turnbull's staining, consistent with previous reports that most iron in the brain is in the Fe^{3+} form (*25*). Both classical histological stains systematically underestimated the iron concentration in the cortex compared to white matter (Fig. S3). This may be due to different staining efficiencies in WM and GM and emphasizes the necessity of quantitative histology for the study of contrast mechanisms in MRI.

The two most important proteins involved in iron storage and iron transport are ferritin and transferrin. It is known that ferritin contains more than 80% of the total tissue iron (*25*, *32*). Interestingly, neither of the two proteins was present in the SWM in higher concentrations than in DWM (not shown), indicating that the elevated iron level in the SWM is more due to a higher iron loading of ferritin cores than to a higher concentration of ferritin proteins. Likewise, the density of microglia was not increased in the SWM (Fig. S4). Instead, microglia was rather homogeneously distributed across the cortex and the WM. A higher density of oligodendrocytes and activated astroglia was observed in the SWM (Fig. S4).

SI.4 Analysis of *in vivo* MRI data

Cortical curvature and thickness maps generated with Freesurfer (Fig S5) were smoothed tangentially to the cortical surface (FWHM 6 mm), resampled to the template surface 'fsaverage' and averaged across subjects to allow comparison with the spatial pattern of the SWM mapping.

SI.5 Estimation of myelin volume fraction from sulfur and phosphor maps measured with LA-ICP-MSI

The myelin volume fraction was estimated based on quantitative maps of sulfur and phosphorus concentrations obtained with LC-ICP-MSI and the method proposed by Stüber *et al.* (*21*). This method assumes that (i) myelin has a constant ratio of sulfur to phosphorus, which is different from that of unmyelinated tissue; (ii) unmyelinated tissue (including proteins, non-lipid macromolecules, DNA) also has a constant ratio of sulfur to phosphorus concentrations; (iii) cortical layer II does not contain any myelin and can be used to determine sulfur to phosphorus ratio of unmyelinated tissue; (iv) myelin volume fraction of white matter is equal to 0.6. Based on these assumptions, the estimation of myelin volume fraction was performed in two steps. First the ratio of sulfur to phosphorus concentrations for unmyelinated tissue was obtained in manually segmented regions-of-interest located in cortical layer II:

$$k_{\text{rest}}=n^{P}_{\text{layer II}}/n^{S}_{\text{layer II}}$$

where $n^{P}_{\text{layer II}}$ and $n^{S}_{\text{layer II}}$ are averaged values of phosphorus and sulfur concentrations obtained with LA-ICP-MSI in specific ROIs. In a second step the myelin volume fraction was calculated:

$$c_{\text{mye}}=n^{P}\text{-}k_{\text{rest}}\,n^{S}$$

, where c_{mye} is the relative myelin volume fraction. The obtained map of myelin fraction was normalized to provide an averaged value of 0.5 in deep white matter (*21*).
